# Toward Antibiotic Stewardship: Route of Antibiotic Administration Impacts the Microbiota and Resistance Gene Diversity in Swine Feces

**DOI:** 10.3389/fvets.2020.00255

**Published:** 2020-05-19

**Authors:** Nicole Ricker, Julian Trachsel, Phillip Colgan, Jennifer Jones, Jinlyung Choi, Jaejin Lee, Johann F. Coetzee, Adina Howe, Susan L. Brockmeier, Crystal L. Loving, Heather K. Allen

**Affiliations:** ^1^Food Safety and Enteric Pathogens Research Unit, ARS-USDA National Animal Disease Center, Ames, IA, United States; ^2^Agricultural and Biosystems Engineering, Iowa State University, Ames, IA, United States; ^3^College of Veterinary Medicine, Kansas State University, Manhattan, KS, United States; ^4^Virus and Prion Research Unit, ARS-USDA National Animal Disease Center, Ames, IA, United States

**Keywords:** antibiotic usage, resistance, microbiome, oxytetracycline, swine

## Abstract

Oral antibiotics are a critical tool for fighting bacterial infections, yet their use can have negative consequences, such as the disturbance of healthy gut bacterial communities and the dissemination of antibiotic residues in feces. Altering antibiotic administration route may limit negative impacts on intestinal microbiota and reduce selective pressure for antimicrobial resistance genes (ARG) persistence and mobility. Thus, a study was performed in pigs to evaluate route of therapeutic oxytetracycline (oxytet) administration, an antibiotic commonly used in the U.S. swine industry, on intestinal microbial diversity and ARG abundance. Given that oral antibiotics would be in direct contact with intestinal bacteria, we hypothesized that oral administration would cause a major shift in intestinal bacterial community structure when compared to injected antibiotic. We further postulated that the impact would extend to the diversity and abundance of ARG in swine feces. At approximately 3 weeks-of-age, piglets were separated into three groups (*n* = 21–22 per group) with two groups receiving oxytet (one via injection and the second via feed) and a third non-medicated group. Oxytet levels in the plasma indicated injected antibiotic resulted in a spike 1 day after administration, which decreased over time, though oxytet was still detected in plasma 14 days after injection. Conversely, in-feed oxytet delivery resulted in lower but less variable oxytet levels in circulation and high concentrations in feces. Similar trends were observed in microbial community changes regardless of route of oxytet administration; however, the impact on the microbial community was more pronounced at all time points and in all samples with in-feed administration. Fecal ARG abundance was increased with in-feed administration over injected, with genes for tetracycline and aminoglycoside resistance enriched specifically in the feces of the in-feed group. Sequencing of plasmid-enriched samples revealed multiple genetic contexts for the resistance genes detected and highlighted the potential role of small plasmids in the movement of antibiotic resistance genes. The findings are informative for disease management in food animals, but also manure management and antibiotic therapy in human medicine for improved antibiotic stewardship.

## Introduction

Antibiotics are a critical tool for fighting bacterial infection in both human and veterinary medicine; yet there is increasing recognition of the need for judicious use of antibiotics to mitigate widespread resistance development. The relative contribution of antibiotic use in food animals to the human antibiotic resistance crisis is poorly defined; however, U.S. regulation on veterinary antibiotic usage has increased in the last few years. Efforts to improve antibiotic stewardship include defining appropriate judicious uses in animal agriculture, for example by disallowing the use of antibiotics in food animals for growth-promotion purposes. Disease treatment and prevention are currently the only approved label uses for antibiotics in food animals in the U.S. (1). Judicious practices may include treating only animals with clinical presentation as opposed to prophylactic or metaphylactic treatment to large numbers of animals.

The swine gastrointestinal microbiota harbors a diverse population of bacteria that play a role in pig health (2–4) but may also be a source of antibiotic resistance genes (ARG) (5). Disturbances to the gut microbiota may enhance ARG transfer and/or enhance abundance of antibiotic resistant bacteria shed from the animal (6, 7). Post-weaning piglets are highly susceptible to a number of diseases, and prophylactic oral antibiotics (in-feed or in-water) are commonly administered to prevent disease (8, 9). It is not uncommon for animals without clinical presentation to be treated with therapeutic antibiotics if other animals in the barn have been diagnosed with bacterial disease. Antimicrobials alter the microbial community throughout the swine gastrointestinal tract [reviewed in (10)]. Antibiotic driven shifts in the swine gastrointestinal microbiota vary in duration, and different taxa shift depending on antibiotic and intestinal segment (7, 11–17). Culture-independent methods (such as qPCR and shotgun metagenomics) are now commonly used to monitor ARG abundance in animal microbiota and the environment (18–20). The development of a common set of primers by Stedtfeld et al. (21) has facilitated the high-throughput analysis of a selection of common ARG across diverse samples. The ability to monitor multiple ARG simultaneously allows for the evaluation of previously unknown co-selection relationships within the microbiome that may influence gene persistence.

Antibiotics remain a necessary tool for limiting disease in food animals (22), and practices to minimize the abundance and persistence of ARGs in swine microbiota, and swine manure applied as fertilizer, is important for both veterinary and human health. Practices that maintain the ability to treat an animal but limit the disturbance to the gastrointestinal microbiota may be one component of antibiotic stewardship. Although oral antibiotic administration is less expensive and more convenient at the herd level, contact with the intestinal bacterial community may drive ARG abundance and mobility. Thus, to provide a method to treat an animal, but limit the impact on intestinal bacteria, we conducted a study to define the impact of injected vs. in-feed delivery of a therapeutic dose of commonly administered antibiotic in swine. The goal of our work was to determine whether the negative impacts of oral antibiotic administration on the gut microbiome, either community disturbance or increased resistance gene abundance, could be mitigated by changing the route of administration to intramuscular injection.

## Materials and Methods

### Sampling Procedures

Animal studies were conducted in accordance with the recommendations in the Guide for the Care and Use of Laboratory Animals. The animal experiments were reviewed and approved by USDA-National Animal Disease Center Animal Care and Use Committee. Ten sows were farrowed in environmentally controlled barns, with 65 piglets weaned at approximately 21 days-of-age and distributed across the three treatment groups to separate littermates (*n* = 21–22/group) (Supplemental Table 1). Two individual pens were established for each treatment in order to evaluate the impact of pen effect on observed differences. Oxytetracycline (oxytet) was used in this study because it is available in both an in-feed and injectable formulation, and is commonly used in the swine industry (23). One group was given oxytet in-feed for 7 days (“Feed” treatment group, Terramycin®100, Phibro) and the in-feed dose was formulated to 10 mg/lb of body weight daily (assuming 11 lb and 450 g feed per pig per day). A second group was given a single intramuscular oxytet injection at 9 mg/lb (“Inject” treatment group, Liquamycin LA-200®, Zoetis), using an estimated weaning weight of 11 lb to calculate injected dose. The third group received no antibiotic and was designated the non-medicated group (“NM” treatment group). Pigs in each group were necropsied on day 4 (*n* = 7/group), 7 (*n* = 7/group), and 14 (*n* = 7–8/group) for collection of ileal and cecal mucosal scrapings. Plasma and feces were collected as previously described (24) at timepoints indicated below to monitor oxytet levels. DNA was isolated from feces and mucosal scrapings for microbiota and ARG analysis (feces only). Feces were collected fresh and transported on ice, aliquoted for downstream applications, and stored at −80°C, as previously described (24). Colon mucosal samples were obtained by gently rinsing 2-inch square sections of proximal colon tissue and then scraping the mucosa with a sterile cell lifter. Scrapings were transported on ice and frozen at −80°C until extraction. DNA was extracted using the PowerMag fecal DNA/RNA extraction kit (MoBio). Body weights were recorded on day 0 and at necropsy.

### Oxytetracycline Concentrations in Tissue

Concentration of oxytetracycline was measured in feces and plasma (days 0, 1, 3, 4, 7, 9, 11, and 14), and intestinal samples collected at necropsy (days 4, 7, and 14) using high-pressure liquid chromatography (Agilent 1100 Pump, Column Compartment and Autosampler, Agilent Technologies, Santa Clara, CA, USA) with mass spectrometry detection (LTQ Ion Trap, Thermo Scientific, San Jose, CA, USA). Samples, spikes, QC's, and blanks (100 μL), were protein precipitated in 1.5 mL microcentrifuge tubes with 400 μL of acetonitrile/0.1% formic acid. An internal standard, demeclocycline, was incorporated into the acetonitrile precipitating agent at a concentration of 200 ng/mL. The samples were vortexed for 5 s after the addition of the acetonitrile and centrifuged for 20 min at 7,500 rpm to sediment the protein pellet. Following centrifugation, the supernatant was poured off into tubes and evaporated to dryness in a Turbovap at 48°C. The tube contents were reconstituted with 150 μL of 8% acetonitrile/0.25% formic acid and transferred to autosampler vials equipped with 300 μL glass inserts. The samples were centrifuged at 2,500 rpm prior to LC-MS analysis.

For LC-MS analysis the injection volume was set to 15 μL. The mobile phases consisted of A: 0.1% formic acid in water and B: 0.1% formic acid in acetonitrile at a flow rate of 0.275 mL/min. The mobile phase began at 5% B with a linear gradient to 95% B in 5.50 min, which was maintained for 1.75 min, followed by re-equilibration to 5% B. Separation was achieved with a HypersilGoldC18 column, 50 mm × 2.1 mm, 1.9 μm particles, Thermo Scientific, San Jose, CA, USA) maintained at 50°C. Oxytet and demeclocycline eluted at 3.43 and 3.82 min, respectively. Full scan MS with wideband activation was used for analyte detection and three fragment ions were used for quantitation of each analyte species. The fragment ions for oxytet were at 398, 408, and 426 m/z, while ions at 289, 430, and 431 m/z were characteristic of demeclocycline fragmentation. Sequences consisting of plasma blanks (porcine plasma), calibration spikes, QC's, and porcine samples were batch processed with a processing method developed in the Xcalibur software (Thermo Scientific, San Jose, CA, USA). The processing method automatically identified and integrated each peak in each sample and calculated the calibration curve based on a weighted (1/X) linear fit. Concentrations of oxytet in unknown samples were calculated by the Xcalibur software based on the calibration curve. Results were then viewed in the Quan Browser portion of the Xcalibur software. Twelve calibration spikes were prepared in blank porcine plasma covering the concentration range of 1 to 5,000 ng/mL. Calibration curves exhibited a correlation coefficient (*r*^2^) exceeding 0.995 across the concentration range. QC samples at 7.5, 75, and 750 ng/mL were within a tolerance of ±15% of the nominal value. The limit of quantitation (LOQ) of the analysis was 2.0 ng/mL with a limit of detection (LOD) of 0.3 ng/mL.

### Microbiome Sequencing and Statistical Analysis

Amplicons of the V4 region of the 16S rRNA gene were generated, sequenced, and analyzed in accordance with the Mothur SOP protocol [(25); https://www.mothur.org/wiki/MiSeq_SOP accessed March 2017], with the addition of removing singletons and doubletons using the split_abund command (cut-off = 2). Sequencing error rate was calculated by sequencing mock communities (26) and was found to be 1.2e-06 errors per basecall. The mothur output was analyzed in R using the phyloseq (27), vegan (28), and DESeq2 (29) packages. The total read counts for the ileal samples were deemed insufficient for further analysis and therefore the 16S bacterial diversity was only evaluated on fecal and colon mucosal samples. Community structure similarity analyses were performed by calculating Bray-Curtis dissimilarities on rarefied OTU tables (3,133 sequences per sample), and statistical testing was accomplished using vegan's adonis function with *post-hoc* comparisons being done with pairwise adonis tests using false discovery rate (FDR) *P*-value correction to account for multiple comparisons. Differential abundance was calculated using the DESeq2 package using Wald tests with parametric fits and FDR-corrected *P*-values. Prior to testing, OTUs with fewer than 10 counts globally were removed and the resulting unrarefied counts were used as the input for DESeq2, as the package recommends. OTUs were agglomerated at various taxonomic levels using phyloseq and these unrarefied agglomerated tables were used as inputs for DESeq2. Phyla level statistical significance was assessed using *T*-tests.

### High Throughput Array-qPCR Analysis

In order to evaluate the impact of antibiotic administration route on ARG abundance within the fecal bacterial communities, DNA from both day 7 and day 14 samples were analyzed by high throughput array-qPCR on the Takara (formerly Wafergen) SmartChip system through Michigan State University using previously validated qPCR primers (21). Primers targeting a total of 48 different genes (resistance or mobility genes, Supplemental Table 2) were analyzed in duplicate. A Ct cutoff value of 28 was applied to all analyses, and the obtained values were analyzed using the delta CT method using 16S as the reference gene (30). In order to determine statistical differences between treatment groups, an ANOVA followed by Tukey's HSD *post hoc* test were performed for each gene. All calculated *P*-values were then corrected by the false discovery rate method.

### Plasmid DNA Isolation and Sequencing

Alkaline lysis plasmid extraction was performed on 10 grams of feces for each animal collected at day 7 (*n* = 7 per group) and followed the protocol of Kav et al. (31) with the following exceptions: only one lysis protocol was used (see Supplemental Methods) and samples consisted of 10 grams of fecal material resuspended in 40 mL of extraction buffer. Neutralization was performed by adding 75 mL of 2 M Tris at pH 7.5 as opposed to adding 60 mL of 2 M Tris at pH 7. Samples were treated with plasmid-safe ATP-dependent DNase (Epicenter) and amplified with Genomiphi DNA polymerase (GE Healthcare) prior to sequencing. Although attempts were made to degrade chromosomal content in these plasmidome samples, comparisons of 16S content before and after treatment indicated that this had variable effectiveness across samples and complete removal of chromosomal DNA was not achieved. Therefore, samples are referred to as plasmid enriched.

Short-read sequencing on each individual plasmidome-enriched sample was performed on the Illumina HiSeq 3000 (paired end and 150 bp high output mode). Sequencing depth ranged from 96 to 288 million reads per fecal sample and sequences were combined to a single assembly that corresponded to the complete plasmidome metagenome. The metagenome assembly pipeline included Trimmomatic v0.36 (32), digital normalization using digiNorm from the khmer package (33) and assembly with Megahit v 1.1.1 (34, 35). Quast v3.1 (36) was used to obtain assembly statistics on the final contigs. This assembly contained 1,877,620 contigs (881,559 contigs larger than 1,000 bp) with a total of 3,784,778,735 bp assembled and an N50 value of 3,653. Assemblies of individual plasmidome metagenomes for each treatment group were also performed using the same pipeline to determine resistance gene diversity by treatment. A single pooled sample was also submitted for long-read sequencing using the Pacific Biosystems RS II system and contigs were assembled using Canu v. 1.6 (37). Resistance genes were identified using the ABRicate software (https://github.com/tseemann/abricate) and Resfinder (38) database (updated 2018-Feb-23) with cut-off values of 98% sequence identity and 80% coverage.

### Data Availability

Data and scripts are available through the Food Safety and Enteric Pathogens Research Unit github site (https://github.com/USDA-ARS-FSEPRU/FS1) and the sequencing data are available through the NCBI SRA (PRJNA553258).

## Results

### Route of Antibiotic Administration Impacted Antibiotic Concentrations in the Pig

**Figure 1:**
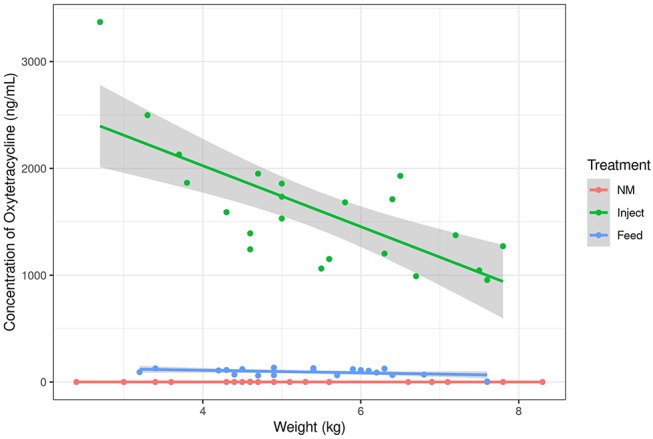
Oxytetracyline concentration in swine plasma samples as measured by LC-MS. Oxytet concentration at day 1 is plotted relative to the animal's weight at initiation of treatment (day 0). The lines correspond to trends for each treatment group. “NM” = Non-medicated, “Inject” = injected oxytet, “Feed” = in-feed oxytet.

**Figure 2:**
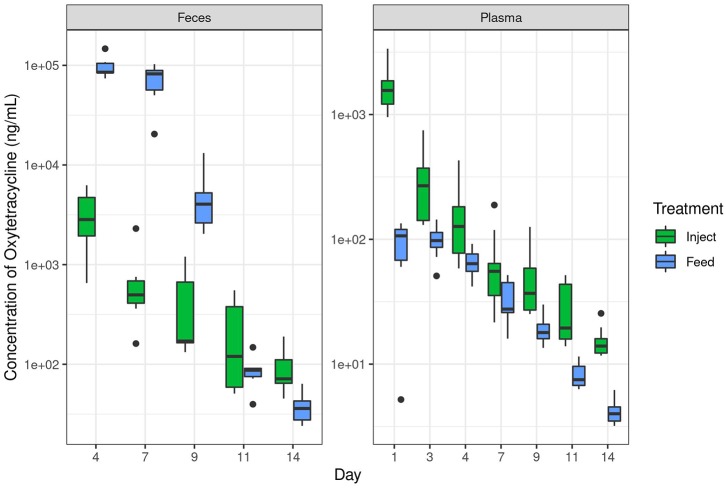
Oxytetracyline levels in swine feces and plasma over time as measured by LC-MS. Samples were collected at the indicated time point and oxytet concentrations determined by LC-MS. The Inject group received a single therapeutic dose on day 0 and the Feed group received therapeutic dose in-feed up to day 7 as described in the methods. Data are plotted on a log_10_ scale. Note the different y-axis for each graph, necessary due to the broad range of concentrations observed in respective compartments. “Inject” = injected oxytet, “Feed” = in-feed oxytet.

Animals in the Inject group had a spike in oxytet concentration the day after injection (day 1) and the Inject group maintained higher plasma oxytet concentrations throughout the course of the experiment compared to animals in the Feed group (Table 1). Weighing individual pigs prior to administration of injected antibiotics would not be feasible in a production setting; therefore, the same dose of oxytet was administered to all pigs in the Inject group. Figure 1 indicates the concentration of oxytet in the plasma on day 1 correlated with pig body weight measured on day 0 (linear regression model, *P* = 0.000119, *R*^2^ = 0.51). Regardless of body weight, oxytet concentrations in the Inject group were an order of magnitude higher than the Feed group (Figure 2). The Inject group plasma concentrations on day 4 was also inversely correlated with weight at day 0 (*P*-value = 0.01122, *R*^2^ = 0.2447; Supplemental Figure 3A); however, at this time point, overall oxytet concentrations in the Inject group were similar to the Feed treatment group (Table 1). Plasma concentrations for the pigs in the Feed group did not correlate with weight and ranged from ~30 to 100 ng/mL during the 7 day course of treatment.

**Table 1:** Mean (+/–SE) oxytet concentration (ng/mL) by treatment for each tissue on day 4 of treatment as measured by LC/MS.

	**Plasma**	**Ileum**	**Fecal**
**Non-Medicated (NM)**	0.1 +/– 0	0 +/– 0	0 +/– 0
**Feed**	66.2 +/– 0.6	4,455.4 +/– 140.2	97,744.5 +/– 3,508.5
**Inject**	151.9 +/– 5.2	240.8 +/– 17.9	3,294.8+/– 283.9

*Day 4 was used for this comparison since tissue samples were only collected at time of necropsy (days 4, 7, and 14). All concentrations are listed in ng/mL, consistent with the standards used for comparison*.

In contrast to plasma levels of oxytet in the Inject group, the ileum and fecal samples contained more oxytet for the Feed group compared to the Inject group (Table 1). Notably, samples from the Feed group had significantly lower oxytet levels in the plasma (mean 66 ng/mL), and instead, oxytet concentrations were much higher in the feces (mean 32,581 ng/mL). The combination of high fecal concentration and low plasma concentration compared to the Inject group illustrate that a substantial portion of the antibiotic received in-feed is directly excreted in fecal waste with limited systemic distribution in the host. Oxytet concentrations in the feces continued to be detected after cessation of treatment. The Feed group continued to have high oxytet (mean 5,030 ng/mL) in feces at day 9 (2 days after withdrawal of medicated feed) but had decreased to 88 ng/mL by day 11 and 38 ng/mL by day 14. The Inject group continued their gradual reduction in excretion, ranging from 454 ng/mL on day 9 to 92 ng/mL by day 14 (Supplemental Table 3B).

### Gut Bacterial Community Is Differentially Impacted by Route of Antibiotic Administration

**Figure 3:**
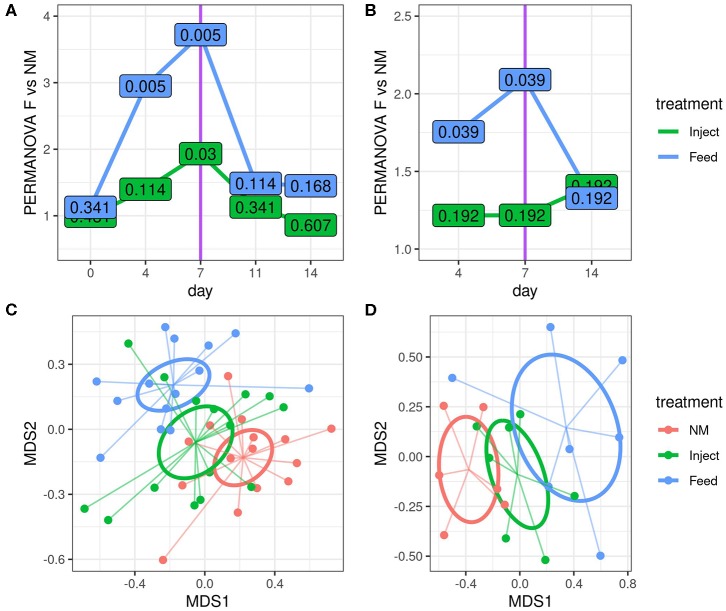
Route of oxytetracyline administration impacted the magnitude of community disturbance relative to the non-medicated (NM) group, as determined by 16S rRNA gene amplicon analysis. Differences in community structure relative to the NM group were calculated using a series of pairwise PERMANOVA tests comparing each treatment to the NM group at indicated time point using Bray-Curtis dissimilarities for both fecal **(A)** and colon mucosa **(B)** communities. The y-axis displays the PERMANOVA pseudo F statistic (total intergroup dissimilarity divided by total intragroup dissimilarity); greater pseudo *F* values indicate greater differences between the group under consideration and the NM group. The values displayed at each point are the corresponding permuted FDR corrected *P*-values for each test. NMDS visualization of Bray-Curtis dissimilarities are also shown for fecal **(C)** and colon mucosa **(D)** microbiota communities on day 7. Ellipses represent the standard error of the centroid for each group.

16S rRNA gene amplicon analysis suggests the community structure of the fecal microbiota was strongly influenced by oxytet route of administration with the Feed group exhibiting the greatest changes relative to the NM group (Figures 3A,C). The microbiota shift was also evident in the Inject group but was only statistically significant at day 7 of study. The community wide changes were also appreciated in colon mucosal samples. Here bacterial communities from the Feed group differed significantly from the NM group at both day 4 and day 7 (Figures 3B,D), but were not different on day 14 (7 days after the end of treatment). Colonic mucosal bacterial community structure was not significantly different between the Inject group and NM group on any of the days evaluated.

**Figure 4:**
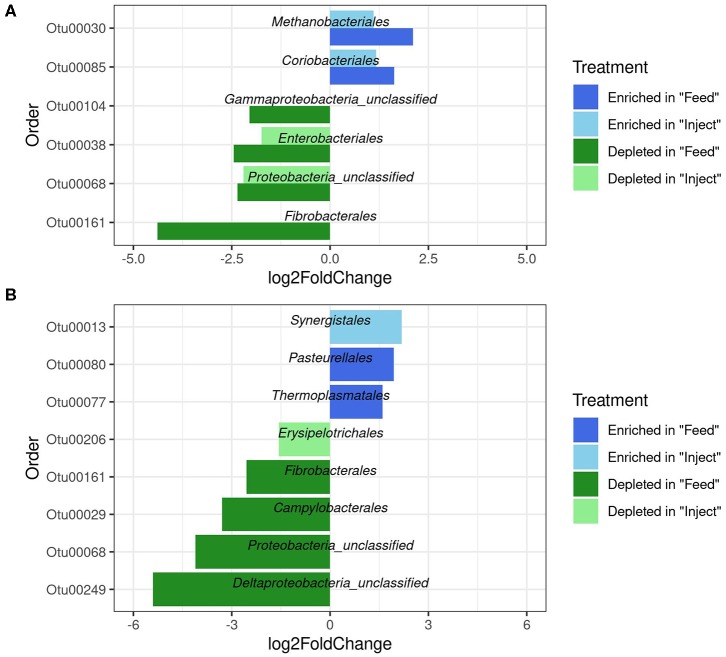
Significantly differentially abundant microbial groups at the Order level (*p* < 0.05) in fecal microbiome after antibiotic administration via Feed or Injection on day 4 **(A)** and day 7 **(B)** via Feed or Injection. All comparisons are to the non-medicated (NM) group of animals.

To investigate how oxytet treatment impacted the abundance of specific bacterial taxa relative to the NM group, the fecal and colon mucosa samples were analyzed at both the phylum and order level at each time point. In the fecal samples, the phyla *Fibrobacteres* and *Proteobacteria* were significantly decreased in the Feed group compared to the NM group at both day 4 and day 7, and significant increases in *Euryarchaeota* and *Actinobacteria* were detected at day 4 within the Feed group relative to the NM group (Supplemental Table 4). Members of the *Actinobacteria* and *Euryarchaeota* phyla were also significantly increased when examining changes in specific orders within the fecal communities; however, the majority of the orders that decreased in abundance in the Feed treatment group belonged to the *Proteobacteria* (Figure 4). No significant difference at the phylum level, regardless of sampling day or location, was detected in feces of the Inject group compared to the NM group. Only two significant decreases at the order level were detected in the Inject group at day 4 (Figure 4A), both of which were members of the *Proteobacteria*. Overall, changes in the Inject treatment group were lower in magnitude and affected fewer orders than the Feed treatment group. The largest fold-change decrease seen in the fecal samples differed by day, with the order *Fibrobacterales* showing a 4-fold decrease relative to NM group animals on day 4 and unclassified *Delta-Proteobacteria* showing a 5-fold decrease at day 7, both of which occurred only in the Feed group (Figure 4B).

**Figure 5:**
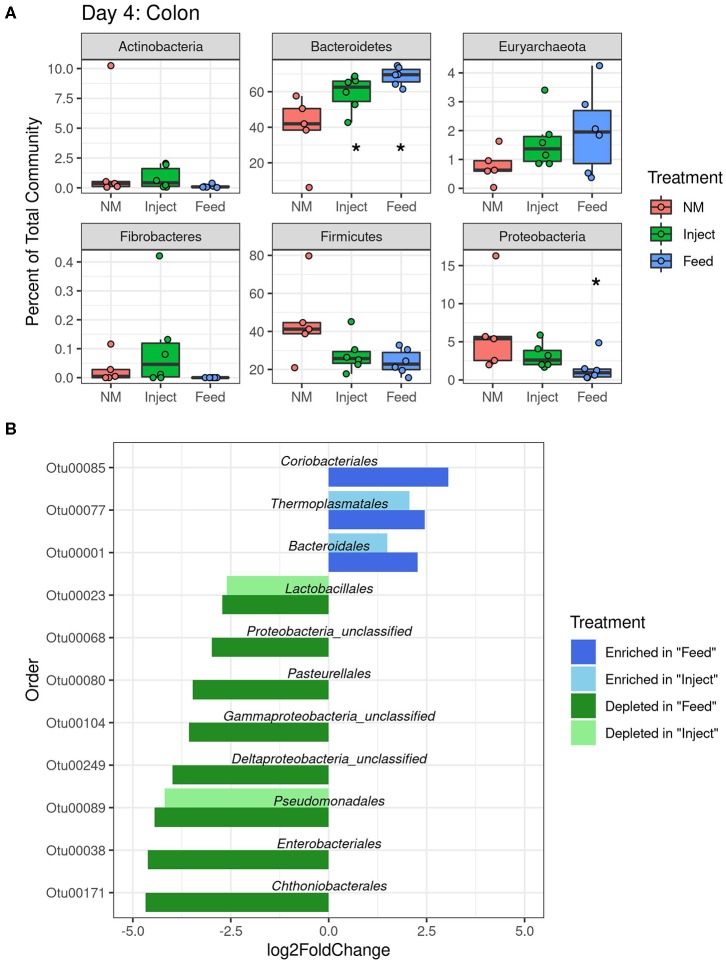
Changes in relative abundance at colon mucosa due to oxytet administration route. Phylum level **(A)** analysis of taxonomic changes at day 4 (*P* < 0.05 indicated with an asterisks). Order level **(B)** changes in abundance (*P* < 0.05) for each treatment group compared to the non-medicated (NM) animals at day 4.

The colonic mucosa community had more significant changes at the phylum level than were seen in the fecal community. Changes in the ratio of *Firmicutes* to *Bacteroidetes* (F:B ratio) were detected in the colon mucosa at day 4 for both oxytet groups (Figure 5A) due to a significant increase in *Bacteroidetes* (Feed to NM *P* = 0.001, Inject to NM *P* = 0.018). At the phyla level, *Proteobacteria* were significantly reduced in the colonic mucosa of the Feed group on day 4 (*P* = 0.032), but not at day 7 or 14. Similar to shifts in feces, the colon mucosa had decreases in several orders of *Proteobacteria* with changes of greater magnitude evident in the Feed group (Figure 5B). There were fewer changes observed at day 7 and 14 in the colon mucosa, and only the *Firmicutes* were significantly impacted in the Inject group at day 14 (*P* = 0.023) (Supplemental Table 4).

### Resistance Gene Abundance Impacted by In-Feed Administration Over Injected

**Figure 6:**
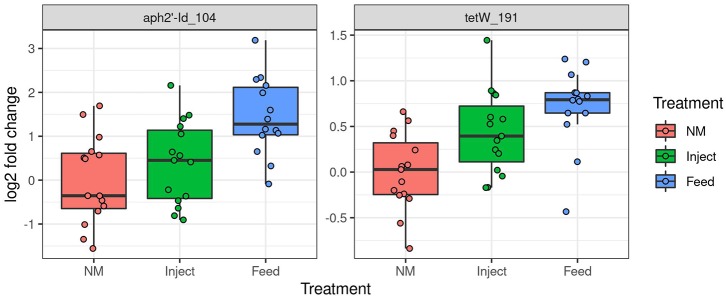
Oxytet administration impacts abundance of antibiotic resistance genes in pig feces. Significant differences in abundance of antibiotic resistance genes in feces on day 7 based on high-throughput qPCR analysis. The Y axis is log2 fold change relative to the mean of the NM group.

Only two ARG had significantly higher prevalence in fecal samples at day 7 (Figure 6)—one encoding tetracycline resistance (primer label *tetW_191*) and the other encoding aminoglycoside resistance (primer label *aph2*′*-id_104*). Abundance of these genes was significantly higher in the Feed treatment compared to the NM group (Tukey's HSD test, FDR adjusted *P* = 0.01 for both) but not significantly higher in the Inject group. Overall prevalence of resistance genes, particularly to tetracyclines, continued to be high for all animals at day 14 regardless of treatment though shifts in abundance were detected through the course of treatment.

In addition to qPCR analysis, fecal plasmid DNA was sequenced using both long-read (Pacific Biosystems) and short-read (Illumina HiSeq) technologies to provide context to the resistance genes present. A master assembly for each technology combining all treatments was screened for matches to the genes found to be increased by qPCR analysis. For *tetW*, there was a single 4 kb contig from the PacBio assembly containing a match to the *tetW* gene as well as genes for a partial type IV secretion system, suggesting it could be part of a conjugative element (39). In addition, there were 14 contigs from the Illumina sequencing with complete or partial matches to the *tetW* gene. The longest of the 14 Illumina contigs was 15,397 bp; however, the closest match in the NCBI GenBank database had only 21% coverage. An examination of the region surrounding the *tetW* gene in our contig had top hits consistent with a plasmid mobilization protein (mob_pre) directly adjacent to *tetW*. Screening the Illumina contigs for the Mob protein confirmed that a small contig containing only the mob protein was also present in the assembly, suggesting multiple genetic contexts for this gene. Similarly, the aminoglycoside gene (*aph2*) identified through qPCR was present on 3 separate contigs in the Illumina assembly, all of which were 1.2 kb or smaller, indicating multiple genetic contexts for the gene which subsequently prevented assembly. The *aph2* gene was not identified in the PacBio assembled contigs. Mapping of the Illumina reads to the assembled contigs confirmed that the resistance genes had high coverage (2,000–5,000 reads per treatment; Supplemental Figure 5) indicating that the fragmented assemblies were not a result of insufficient coverage.

There was low overall prevalence of resistance genes within the PacBio assembled contigs; however, there were two resistance plasmids assembled that may give some insight into the transfer dynamics of both tetracycline and aminoglycoside genes within the swine microbiome. Both contigs are 6 kb in size, carry the same plasmid backbone and carry distinct antibiotic resistance genes that were acquired in separate locations in the plasmid. Small plasmids, lacking any AMR genes, highly similar to the aforementioned plasmids were also identified in the assembly, suggesting a diverse population of small plasmids within the swine microbiome.

## Discussion

Oral antibiotic administration to pigs causes significant disturbances to intestinal microbial community, dependent on the antibiotic administered (10–13, 15–17, 40, 41). Disturbances are not limited to the bacteria in intestinal lumen (represented by fecal analysis), but also shift bacterial populations at the intestinal mucosa. Alterations to the structure of the bacterial community can have important implications for host metabolism (9, 15, 42, 43) as well as providing opportunities for the establishment of specific pathogens (44, 45). In a recent study, (46) specifically evaluated the impact of parenteral injection of five different antimicrobials currently used in swine production and identified antimicrobial-specific shifts in the microbial community during the course of treatment. Furthermore, a study looking at oxytetracycline administration in mice illustrated the differential impact of route of administration on the colonization and persistence of bacteria carrying resistance genes to the administered antibiotic (47). In this work we have expanded on these previous studies by evaluating the differential impact of a single antibiotic administered by two different routes at therapeutic level and evaluated these changes on the complete microbial community as opposed to an introduced strain.

Many factors drive shifts in the intestinal microbiota, including time, diet, and antimicrobials (48). In this study, the covariate with the greatest influence on microbial community structure was time, which correlated with dietary change of weaning to solid food (13). Although the time-driven shift in the community complicated analysis, the post-weaning time period was important to our experimental design since antibiotics are often administered at this stage to prevent post-weaning diarrhea. Antibiotic treatment caused similar changes in microbial communities regardless of route of administration; however, the impact on the microbial community was more pronounced at all time points and in all samples with in-feed administration. Decreased Proteobacteria at days 4 and 7 after treatment was a somewhat unexpected result, both due to the prevalence of tetracycline resistance in *E. coli* (a common member of the Proteobacteria) in swine, and published studies observing increases in *E. coli* abundance following antibiotic administration (7, 12, 16). Specifically, a recent study on oxytetracycline in swine (7) identified increases in abundance of *Escherichia/Shigella* OTU's in response to tetracycline treatment on day 8 after treatment. Our analysis was performed at the Order level as opposed to the OTU level, and therefore speaks to a broader impact on the Proteobacteria that may not be reflected in individual genera. Examining our data at the OTU level, the only significant change in the *Escherichia/Shigella* OTU was a decrease on day 4 at the colon mucosa (*P* = 4.8 × 10^−6^) and an increase in the feces at day 7 (*P* = 0.03), both of which occurred solely in the Feed treatment group (data not shown). Therefore, although there was an overall decrease in Proteobacteria observed at day 4 and day 7, *E. coli* abundance in feces was increased toward the end of oral oxytet treatment, consistent with other studies.

The decrease in endogenous Proteobacteria populations may have negative consequences for the host's resistance to colonization by opportunistic pathogens. In a recent study, Velazquez et al. demonstrate that endogenous *Enterobacteraceae* populations play a critical role in determining susceptibility to *Salmonella* colonization and infection in mice. *Enterobacteraceae* populations compete with *Salmonella* for the terminal electron acceptors that drive their respiratory metabolisms (49). Many important foodborne pathogens such as *E. coli, Salmonella*, and *Campylobacter* utilize respiratory metabolisms (50) and as endogenous (benign) populations using terminal electron acceptors are depleted, the compounds become available and can be used by various foodborne pathogens to assist in colonization of the host. While this hypothesis needs more rigorous investigation in other host species, there is some evidence that it occurs in pigs, as tetracycline treatment can increase *Salmonella* shedding from pigs (45). Our work suggests that the depletion of endogenous Proteobacteria may be unintended collateral damage with potential negative consequences for the host and that this collateral damage to the gut ecosystem may be mitigated by injecting oxytet as opposed to administering oxytet orally.

Bacterial community shifts give insight into the impact of antibiotic treatment on the overall swine gut community and disturbances may serve as a proxy for factors impacting intestinal health and ARG transfer; however, changes in resistance gene abundance can occur independent of community member shifts due to the selective elimination of susceptible community members (51) and potential horizontal ARG transfer within the community (6). Changes in ARG content separate from taxonomical distribution would be expected to be particularly relevant when the *Proteobacteria* are impacted, as this phyla carries the greatest diversity of mobile ARGs (52). We chose to examine changes in resistance gene abundance specifically in the fecal samples as it evaluates the resistome of the individual animals in a manner amenable to surveillance of fecal resistance genes that could be disseminated to the environment through field application of animal manure.

As noted above, tetracycline resistance in swine *E. coli* is highly prevalent, ranging from 79 to 100% of isolates (53–55). The observed decreases in Proteobacteria can therefore be expected to correlate solely with the tetracycline susceptible members of the community. Tetracycline resistance is commonly carried on plasmids and other mobile elements (56) and the role of plasmids in disseminating ARG has important implications to the overall risk of resistance gene evolution and spread [reviewed in (57)]. Genes associated with tetracycline resistance were more prevalent in feces of animals given oral oxytet, when compared to injected oxytet. There was also a significant increase in abundance of a gene involved in aminoglycoside resistance in feces of the Feed group, suggesting co-selection for bacteria with the gene. This is consistent with previous work highlighting an increase in aminoglycoside resistance with the use of unrelated antibiotics (12). The aminoglycoside gene identified in this case has been documented as transferring between *Enterococcus* and *E. coli* (58). Many ARG were detected in fecal DNA, even in the NM group (data available at https://github.com/USDA-ARS-FSEPRU/FS1/blob/master/wafergen_reanalysis_Oct2018.R), as noted in previous studies (16, 19). However, plasmid specific targets were not detected in any of the fecal DNA samples which indicates that we did not have robust detection of Gram-negative plasmids within the fecal community of these samples. This is also evident in the tetracycline resistance genes detected, as *tetB* was detected at low levels across all of the samples in contrast to *tetM* and *tetW* that were found in high abundance (and are more commonly associated with the Gram-positive strains that dominated the microbiota). Another limitation of using qPCR for resistome analysis is that the genetic context of the ARG cannot be determined. In order to address this limitation, plasmidome enrichment of the fecal samples was performed and the samples sequenced using both long- and short-read technologies. Although these methods gave only limited insight into the genetic context of the genes highlighted in the qPCR analysis, the detection of small (<7 kb) mobilizeable plasmids carrying tetracycline and aminoglycoside resistance genes provides a potential route of dissemination that has been underexplored. The possible role of small plasmids as gene capture platforms has also been identified by other researchers recently and merits further investigation (59–61).

The optimal route of administration of oxytetracycline for therapy may be dependent on the targeted pathogen. Both in-feed and injectable oxytet are labeled for the treatment of bacterial pneumonia caused by *Pasteurella multocida* and bacterial enteritis caused by *Escherichia coli*, though it's unclear if efficacy against each organism is the same regardless of administration route. Plasma concentrations of oxytet following injection were in agreement with previous reports for this formulation (62–64) and likewise, the low absorption of oxytet into circulation after oral administration has been documented (65, 66). Oral oxytet administration to pigs may therefore be more effective against intestinal pathogens, as oral administration resulted in increased exposure of gastrointestinal bacteria to antibiotic, and large amounts of antibiotic in feces. However, it does require an animal to consume feed (or water), and anorexia during illness may limit uptake. Concentrations of OTC in nasal wash were higher in the Feed treatment group, likely as a result of the rooting behavior of swine, and this may provide increased protection against respiratory pathogens but also apply selective pressure for ARG in the nasal bacterial populations (67). For this reason, our group is currently investigating the effectiveness of each of these administration routes against a respiratory pathogen challenge and impact on respiratory microbiota. While we administered oxytet to non-infected pigs, and pharmacokinetics may differ during disease, oxytet is often administered to healthy animals when prophylactic treatment is initiated.

Route of antibiotic excretion and withdrawal times may be another consideration in selecting route of administration, as antibiotic contact with bacteria in the environment is also an important consideration related to resistance. Oxytet in feces ends up in manure pits, which may be spread onto fields and subsequently increase the diversity and abundance of ARG in both the treated animals and soils receiving manure from these animals (7, 18, 20, 68, 69). The bioavailability of oxytetracycline is dependent on the soil structure (35, 70); however, exposure in soil can impact microbial enzyme activity (71), functional microbial community structure (72), and increase the persistence of resistance genes following field application of manure (73). While injected administration led to less oxytet in feces, the amount of oxytet excreted in urine was not measured in the current study and may be the primary excretion site after injection. Between 40 and 60% of intravenous administered oxytet is excreted in urine (62, 74), and this is an important consideration for limiting oxytet in the environment. To limit oxytet residue in meat, oxytet must be removed at least 5 or 28 days prior to slaughter for in-feed and injected administration, respectively. Future studies examining all possible excretion routes would be beneficial to antibiotic stewardship efforts by identifying the optimal administration method to maximize the therapeutic effect of treatment while also minimizing unwanted side-effects, such as the release of antibiotic residues to the environment or the disruption of the healthy microbial communities. Collectively, many factors need consideration for treatment of animals with therapeutic oxytet.

Our results have important implications for antibiotic use in both food production animals and potentially also in human patients. In this study, the impacts of oxytet on the overall gut community, and on abundance of resistance genes, was reduced when the antibiotic was delivered by intramuscular injection as opposed to in-feed. In addition, the amount of oxytet in feces was high with in-feed administration and is an important consideration for selective pressure in the environment. Route of antibiotic administration may therefore be one critical control point for maintaining healthy gut communities and reducing selection for antibiotic resistance genes.

## Data Availability Statement

The datasets generated for this study can be found in the NCBI SRA (PRJNA553258).

## Ethics Statement

The animal study was reviewed and approved by USDA-National Animal Disease Center Animal Care and Use Committee.

## Dedication

This article is dedicated to the memory of Heather K. Allen.

## Author Contributions

NR, JT, AH, SB, CL, and HA designed and performed the experiments. NR, JT, PC, and JCh performed bioinformatic analyses. JT performed statistical analysis and generated figures. JJ and JL performed sequencing and qPCR preparation. JCo performed oxytetracycline analysis. NR, JT, CL, and HA wrote the manuscript. All authors read and approved the manuscript prior to submission. HA passed away on March 7, 2020 while the paper was under review.

## Conflict of Interest

The authors declare that the research was conducted in the absence of any commercial or financial relationships that could be construed as a potential conflict of interest.
