# Supplementary material for: Toward Antibiotic Stewardship: Route of Antibiotic Administration Impacts the Microbiota and Resistance Gene Diversity in Swine Feces
Source: Front Vet Sci. 2020 May 19;7:255. doi: 10.3389/fvets.2020.00255 (PMC7249142; doi:10.3389/fvets.2020.00255)
Supplement: Supplementary file 1 [file Data_Sheet_1.docx]

Supplemental Tables and Figures:

S1. Metadata of animals used in this trial

| **Pig ID Numbers** | **Sow** | **Birth Room** | **DOB** | **Sex** | **Weight (Kg)** | **Necropsy day** | **Control/treatment** | **Pen** |
| --- | --- | --- | --- | --- | --- | --- | --- | --- |
| 1 | 1208 | 1 | 1/29/16 | M | 5.6 | 4 | Control diet | 1A |
| 43 | 1206 | 1 | 1/31/16 | M | 2.4 | 14 | Control diet | 1A |
| 2 | 1211 | 2 | 1/30/13 | M | 3 | 4 | Control diet | 1A |
| 3 | 1207 | 2 | 1/28/16 | M | 8.3 | 4 | Control diet | 1A |
| 4 | 1212 | 3 | 1/31/16 | M | 4.7 | 4 | Control diet | 1A |
| 22 | 1212 | 3 | 1/31/16 | M | 5.6 | 7 | Control diet | 1A |
| 23 | 1209 | 3 | 1/30/16 | F | 7.1 | 7 | Control diet | 1A |
| 44 | 1212 | 3 | 1/31/16 | F | 4.5 | 14 | Control diet | 1A |
| 45 | 1209 | 3 | 1/30/16 | M | 7.8 | 14 | Control diet | 1A |
| 24 | 1215 | 4 | 1/27/16 | M | 6.6 | 7 | Control diet | 1A |
| 46 | 1215 | 4 | 1/27/16 | M | 6.9 | 14 | Control diet | 1A |
| 28 | 1211 | 2 | 1/30/13 | F | 3.6 | 7 | Control diet | 1B |
| 50 | 1211 | 2 | 1/30/16 | M | 5.1 | 14 | Control diet | 1B |
| 5 | 1209 | 3 | 1/30/16 | M | 7.6 | 4 | Control diet | 1B |
| 6 | 1215 | 4 | 1/27/16 | F | 4.3 | 4 | Control diet | 1B |
| 7 | 1214 | 4 | 1/30/16 | F | 3.4 | 4 | Control diet | 1B |
| 25 | 1214 | 4 | 1/30/16 | M | 5.3 | 7 | Control diet | 1B |
| 47 | 1214 | 4 | 1/30/16 | F | 4.4 | 14 | Control diet | 1B |
| 26 | 1210 | 5 | 1/27/16 | F | 4.6 | 7 | Control diet | 1B |
| 27 | 1213 | 5 | 1/29/16 | M | 4.6 | 7 | Control diet | 1B |
| 48 | 1210 | 5 | 1/27/16 | M | 6.9 | 14 | Control diet | 1B |
| 49 | 1213 | 5 | 1/29/16 | M | 4.9 | 14 | Control diet | 1B |
| 10 | 1208 | 1 | 1/29/16 | M | 2.7 | 4 | IM – OTC | 2A |
| 51 | 1208 | 1 | 1/29/16 | F | 7.6 | 14 | IM – OTC | 2A |
| 52 | 1206 | 1 | 1/31/16 | F | 3.8 | 14 | IM – OTC | 2A |
| 11 | 1211 | 2 | 1/30/13 | F | 4.6 | 4 | IM – OTC | 2A |
| 29 | 1212 | 3 | 1/31/16 | F | 5.6 | 7 | IM – OTC | 2A |
| 53 | 1212 | 3 | 1/31/16 | F | 5.5 | 14 | IM – OTC | 2A |
| 54 | 1209 | 3 | 1/30/16 | M | 7.2 | 14 | IM – OTC | 2A |
| 30 | 1214 | 4 | 1/30/16 | M | 4.6 | 7 | IM – OTC | 2A |
| 8 | 1210 | 5 | 1/27/16 | M | 5.8 | 4 | IM – OTC | 2A |
| 9 | 1213 | 5 | 1/29/16 | F | 5 | 4 | IM – OTC | 2A |
| 31 | 1210 | 5 | 1/27/16 | F | 3.3 | 7 | IM – OTC | 2A |
| 33 | 1208 | 1 | 1/29/16 | F | 6.7 | 7 | IM – OTC | 2B |
| 12 | 1207 | 2 | 1/28/16 | F | 7.8 | 4 | IM – OTC | 2B |
| 34 | 1207 | 2 | 1/28/16 | M | 7.5 | 7 | IM – OTC | 2B |
| 13 | 1212 | 3 | 1/31/16 | F | 5 | 4 | IM – OTC | 2B |
| 14 | 1209 | 3 | 1/30/16 | F | 4.3 | 4 | IM – OTC | 2B |
| 35 | 1209 | 3 | 1/30/16 | F | 6.5 | 7 | IM – OTC | 2B |
| 55 | 1215 | 4 | 1/27/16 | F | 6.3 | 14 | IM – OTC | 2B |
| 56 | 1214 | 4 | 1/30/16 | M | 6.4 | 14 | IM – OTC | 2B |
| 32 | 1213 | 5 | 1/29/16 | F | 5 | 7 | IM – OTC | 2B |
| 57 | 1210 | 5 | 1/27/16 | M | 4.7 | 14 | IM – OTC | 2B |
| 58 | 1213 | 5 | 1/29/16 | M | 3.7 | 14 | IM – OTC | 2B |
| 37 | 1208 | 1 | 1/29/16 | M | 6.8 | 7 | In-feed -OTC | 3A |
| 59 | 1208 | 1 | 1/29/16 | F | 5.9 | 14 | In-feed -OTC | 3A |
| 61 | 1206 | 1 | 1/31/16 | F | 5.4 | 14 | In-feed -OTC | 3A |
| 38 | 1211 | 2 | 1/30/13 | M | 4.9 | 7 | In-feed -OTC | 3A |
| 60 | 1211 | 2 | 1/30/13 | M | 5.4 | 14 | In-feed -OTC | 3A |
| 15 | 1215 | 4 | 1/27/16 | F | 6.1 | 4 | In-feed -OTC | 3A |
| 16 | 1214 | 4 | 1/30/16 | F | 3.2 | 4 | In-feed -OTC | 3A |
| 36 | 1215 | 4 | 1/27/16 | M | 6.2 | 7 | In-feed -OTC | 3A |
| 17 | 1210 | 5 | 1/27/16 | M | 3.4 | 4 | In-feed -OTC | 3A |
| 18 | 1213 | 5 | 1/29/16 | M | 4.7 | 4 | In-feed -OTC | 3A |
| 19 | 1208 | 1 | 1/29/16 | F | 5 | 4 | In-feed -OTC | 3B |
| 20 | 1211 | 2 | 1/30/13 | M | 4.5 | 4 | In-feed -OTC | 3B |
| 21 | 1207 | 2 | 1/28/16 | M | 7.6 | 4 | In-feed -OTC | 3B |
| 39 | 1207 | 2 | 1/28/16 | M | 6 | 7 | In-feed -OTC | 3B |
| 40 | 1212 | 3 | 1/31/16 | M | 4.3 | 7 | In-feed -OTC | 3B |
| 41 | 1214 | 4 | 1/30/16 | M | 4.4 | 7 | In-feed -OTC | 3B |
| 63 | 1215 | 4 | 1/27/16 | F | 6.4 | 14 | In-feed -OTC | 3B |
| 64 | 1214 | 4 | 1/30/16 | F | 4.2 | 14 | In-feed -OTC | 3B |
| 42 | 1213 | 5 | 1/29/16 | F | 5.7 | 7 | In-feed -OTC | 3B |
| 65 | 1210 | 5 | 1/27/16 | M | 6.3 | 14 | In-feed -OTC | 3B |
| 66 | 1213 | 5 | 1/29/16 | M | 4.9 | 14 | In-feed -OTC | 3B |

S2. Primer targets for high throughput qPCR. Primers were based on previous work (Stedtfeld et al. 2018)

| Gene Targets | Antibiotic/MGE Class |
| --- | --- |
| aac(6), aphA3, ant6-ia, ant6-ib, aph(2’)-id | Aminoglycoside |
| ermB, ermG, ermA | MLSB |
| tetB, tetG, tetM, tetO, tetQ, tetW, tet32, tet40, tet44, tetK, tetL, tetX | Tetracycline |
| oqxA | Fluoroquinolone |
| blaROB, cfxA, blaCTX-M | Beta-lactam |
| sul2 | Sulfonamide |
| IncHI2, IncI1, IncN, IncP, IncQ, IncF_FIC, IncP-1α, IncP-1β | Plasmids |
| intI1, intI2, intI3, IS26, ISEcp1, tnpA_IS6100, mobA, tnpA_Tn3, IS91, ISCR1 | MGEs |

S3a. Oxytet concentrations on day 4 plotted according to weight of each pig on day 0 (prior to treatment).

S3b. Oxytet concentration in tissue and fecal samples over time

| Day | Tissue | Treatment | Mean | Number of animals | Standard Error |
| --- | --- | --- | --- | --- | --- |
| 4 | Ileum | NM | 0 | 7 | 0 |
| 4 | Ileum | Inject | 240.7857 | 7 | 17.94663 |
| 4 | Ileum | Feed | 4455.457 | 7 | 140.1823 |
| 7 | Ileum | NM | 0 | 7 | 0 |
| 7 | Ileum | Inject | 54.38571 | 7 | 5.980384 |
| 7 | Ileum | Feed | 793.1667 | 6 | 73.31832 |
| 14 | Ileum | NM | 5 | 8 | 1.157275 |
| 14 | Ileum | Inject | 12.5 | 8 | 1.293873 |
| 14 | Ileum | Feed | 11.42857 | 7 | 1.527207 |
| 4 | feces | NM | 0 | 7 | 0 |
| 4 | feces | Inject | 3294.786 | 7 | 283.8604 |
| 4 | feces | Feed | 97744.53 | 7 | 3508.549 |
| 7 | feces | NM | 0 | 7 | 0 |
| 7 | feces | Inject | 738.0429 | 7 | 102.0624 |
| 7 | feces | Feed | 71412.68 | 6 | 5082.211 |
| 9 | feces | NM | 15 | 8 | 1.157275 |
| 9 | feces | Inject | 453.8857 | 7 | 58.71272 |
| 9 | feces | Feed | 5029.671 | 7 | 553.7008 |
| 11 | feces | NM | 17.5 | 8 | 0.883883 |
| 11 | feces | Inject | 217.8125 | 8 | 24.67997 |
| 11 | feces | Feed | 87.63333 | 6 | 5.867932 |
| 14 | feces | NM | 20 | 9 | 0 |
| 14 | feces | Inject | 91.9125 | 8 | 5.987093 |
| 14 | feces | Feed | 37.98571 | 7 | 2.021053 |

S4. Significant taxa changes at the phyla level

| Day | Tissue | Phylum | Group1 | Group2 | p.value |
| --- | --- | --- | --- | --- | --- |
| 4 | Colon | Bacteroidetes | Feed | NM | 0.001 |
| 4 | Colon | Bacteroidetes | Inject | NM | 0.018 |
| 4 | Colon | Firmicutes | Feed | NM | 0.021 |
| 4 | Colon | Proteobacteria | Feed | NM | 0.032 |
| 7 | Colon | Euryarchaeota | Feed | NM | 0.032 |
| 14 | Colon | Firmicutes | Inject | NM | 0.023 |
| 14 | Colon | Bacteria_unclassified | Feed | Inject | 0.041 |
| 4 | Feces | Euryarchaeota | Feed | NM | 0 |
| 4 | Feces | Proteobacteria | Feed | NM | 0.001 |
| 4 | Feces | Actinobacteria | Feed | NM | 0.01 |
| 4 | Feces | Euryarchaeota | Feed | Inject | 0.022 |
| 4 | Feces | Bacteria_unclassified | Feed | Inject | 0.025 |
| 7 | Feces | Proteobacteria | Feed | NM | 0.003 |
| 7 | Feces | Spirochaetae | Feed | NM | 0.015 |
| 7 | Feces | Proteobacteria | Feed | Inject | 0.018 |
| 14 | Feces | Firmicutes | Feed | NM | 0.006 |
| 14 | Feces | Bacteroidetes | Feed | NM | 0.025 |
| 14 | Feces | Bacteroidetes | Inject | NM | 0.037 |

S5. Read coverage for tetW. The tetracycline corresponds with the first high coverage region at the start of the contig (1800 bp in length) and the remainder of the contig had no significant hits by blast to the NCBI non-redundant database.


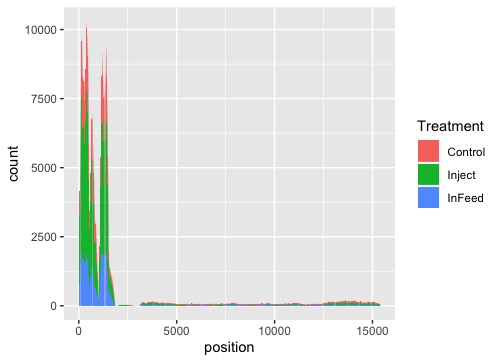


Supplemental methods

**Protocol for isolating plasmids from swine feces**

**DAY 1**

Prep: Make sure rotors are at 4C.

1. Mass out approximately 10 g of fecal material.
2. Place in blender bucket with 35-40 ml extraction buffer (100 mM Tris-HCl, 10 mM EDTA, 0.15 M NaCl, pH 8.0).
3. Blend for 2 min on high speed (NOTE: Pending protocol progress, consider decreasing the blending step. For phage isolation protocol, I only blended for 30 seconds).
4. Pour into a 50 ml conical tube (blue capped falcon tube), incubate on ice for 1 hr.
5. Balance tubes. Centrifuge at 500g for 15 min at 4C.
   1. The resulting pellet is junk
6. Carefully pour the supernatant into a new centrifuge bottle (purple capped tube) through cheesecloth, being careful to avoid the pellet. Balance tubes.
7. Centrifuge at 10,000g for 25 min at 4C.
8. Pellet can be stored at -20C until plasmid DNA extraction.

# **Day 2**

1. Resuspend pellet in 81 mL of resuspension solution (25% sucrose in 50 mM Tris pH8).

2. **Vortex** in order to get clumps into solution.

3. Add 6 mL lysozyme (10 mg/mL in 250 mM Tris pH8).

4. Mix by inversion; Incubate on ice for 5 minutes.

5. Add 30 mL of 250 mM EDTA pH 8. Mix by several inversions. Incubate on ice for 5 minutes.

6. Add 30 mL of 20% SDS in TE. Mix by inversion.

7. Perform 8 cycles of heat pulse - 15 seconds in 55^o^C water bath; 15 sec mixing at room temp.

8. Add 30 mL of 3M NaOH **(must be freshly prepared and precise (volumetric flask))**

9. Mix **immediately** by inversion for 3 minutes.

10. **Add ~75 mL of 2 M Tris pH 7.5 (check pH with strip to ensure below 9.0)**

11. Add 39.6 mL of 20% SDS in TE and **immediately** add 75 mL 5M NaCl.

(Hanson protocol indicates that failure to add and mix quickly at ambient temperature gave incomplete removal of chromosomal DNA)

12. Mix by inversion and chill in a 4^o^C water bath. Incubate 6 hours or overnight at 4^o^C

Day 3:

1. Centrifuge at 10,000 xg for 30 minutes at 4^o^C. Decant supernatant to chilled tube.

If some precipitate remains in solution, either decant supernatant to fresh tube and repeat spin or remove floating precipitate using a pipette.

2. **To supernatant, add 0.313 volume PEG solution (42% wt/vol in 0.01 M sodium phosphate buffer, pH 7,** for a final concentration of 10% PEG**) (Hanson protocol)** - can be modified as appropriate

3. Mix by stirring with a plastic pipette (Hanson method) or by gentle inversion.

**Incubate at 4^o^C overnight or at -20^o^C for up to 3 months**

Day 4:

1. Centrifuge at 16,000 xg for 30 minutes at 4^o^C.

2. Resuspend in water or Tris buffer.

## **Recipes for solutions**

**Resuspension buffer**

25 mL 1M Tris pH8

125 g sucrose

350 mL dH_2_O

Bring up to 500 mL with dH_2_O. Autoclave to sterilize. Store at 4^o^C (or room temperature).

**Lysozyme (10 mg/mL)**

12.5 mL of 250 mM Tris pH 8

500 mg lysozyme

37.5 mL dH_2_O

Aliquot into individual tubes of volume to be used and store at -20^o^C.

**250 mM EDTA pH 8**

Dilute from 0.5M stock and filter to sterilize. Store at room temperature.

**20% SDS in TE**

200 g SDS

600 mL 1X TE

Heat until dissolved and then bring up to 1 L with TE. Store at 37^o^C.

**3 M NaOH**

150 g NaOH pellets

700 mL dH_2_O

Add dH_2_O to 1000 mL (**in a glass bottle?**)

**5M NaCl**

146 g NaCl

350 mL dH_2_O

Add dH_2_O to 500 mL.

**2M Tris pH 7**

242 g Tris-base

750 mL dH_2_O

Adjust pH with HCl (lots). Autoclave and store at room temperature.
